# Hypoxic Preconditioning Attenuates Reoxygenation-Induced Skeletal Muscle Dysfunction in Aged Pulmonary TNF-α Overexpressing Mice

**DOI:** 10.3389/fphys.2018.01720

**Published:** 2018-12-21

**Authors:** Chia-Chen Chuang, Tingyang Zhou, I. Mark Olfert, Li Zuo

**Affiliations:** ^1^Radiologic Sciences and Respiratory Therapy Division, School of Health and Rehabilitation Sciences, The Ohio State University College of Medicine, The Ohio State University Wexner Medical Center, Columbus, OH, United States; ^2^Interdisciplinary Biophysics Graduate Program, The Ohio State University, Columbus, OH, United States; ^3^Division of Exercise Physiology, West Virginia University School of Medicine, Morgantown, WV, United States; ^4^Department of Biology, The University of Maine, Presque Isle, ME, United States

**Keywords:** COPD, diaphragm, hypoxia, preconditioning, reactive oxygen species

## Abstract

**Aim:** Skeletal muscle subjected to hypoxia followed by reoxygenation is susceptible to injury and subsequent muscle function decline. This phenomenon can be observed in the diaphragm during strenuous exercise or in pulmonary diseases such as chronic obstructive pulmonary diseases (COPD). Previous studies have shown that PO_2_ cycling or hypoxic preconditioning (HPC), as it can also be referred to as, protects muscle function via mechanisms involving reactive oxygen species (ROS). However, this HPC protection has not been fully elucidated in aged pulmonary TNF-α overexpressing (Tg^+^) mice (a COPD-like model). We hypothesize that HPC can exert protection on the diaphragms of Tg^+^ mice during reoxygenation through pathways involving ROS/phosphoinositide 3-kinase (PI3K)/protein kinase B (Akt)/extracellular signal regulated kinase (ERK), as well as the downstream activation of mitochondrial ATP-sensitive potassium channel (mitoK_ATP_) and inhibition of mitochondrial permeability transition pore (mPTP).

**Methods:** Isolated Tg^+^ diaphragm muscle strips were pre-treated with inhibitors for ROS, PI3K, Akt, ERK, or a combination of mitoK_ATP_ inhibitor and mPTP opener, respectively, prior to HPC. Another two groups of muscles were treated with either mitoK_ATP_ activator or mPTP inhibitor without HPC. Muscles were treated with 30-min hypoxia, followed by 15-min reoxygenation. Data were analyzed by multi-way ANOVA and expressed as means ± SE.

**Results:** Muscle treated with HPC showed improved muscle function during reoxygenation (*n* = 5, *p* < 0.01). Inhibition of ROS, PI3K, Akt, or ERK abolished the protective effect of HPC. Simultaneous inhibition of mitoK_ATP_ and activation of mPTP also diminished HPC effects. By contrast, either the opening of mitoK_ATP_ channel or the closure of mPTP provided a similar protective effect to HPC by alleviating muscle function decline, suggesting that mitochondria play a role in HPC initiation (*n* = 5; *p* < 0.05).

**Conclusion:** Hypoxic preconditioning may protect respiratory skeletal muscle function in Tg^+^ mice during reoxygenation through redox-sensitive signaling cascades and regulations of mitochondrial channels.

## Introduction

Chronic obstructive pulmonary diseases (COPD) are the fourth leading cause of death in the world, with particularly high prevalence among the elderly population (Cazzola et al., 2010; Huang et al., 2013). The diaphragm shows mechanical overloading in COPD, resulting in progressive muscle weakness and compromised diaphragmatic muscle performance (Orozco-Levi et al., 2001; Debska et al., 2002; Kim et al., 2008). The provision of supplemental oxygen is effective for managing patients’ symptoms, but reoxygenation can exacerbate reactive oxygen species (ROS) production, leading to further muscle damage by impairing contractility and accelerating fatigue (Debska et al., 2002; Stoller et al., 2010; Tang et al., 2013; Zuo et al., 2014b, 2015b; Steinbacher and Eckl, 2015).

In wild-type mice, PO_2_ cycling/hypoxic preconditioning (HPC) exhibits a protective effect on diaphragmatic skeletal muscle by reducing intracellular ROS production during both hypoxia and reoxygenation, as well as improving muscle function (Zuo et al., 2015a,b). It is likely that HPC triggers upstream mediators such as intracellular ROS, phosphoinositide 3-kinase (PI3K), protein kinase B (Akt), and extracellular signal regulated kinase (ERK). HPC also potentially facilitates the opening of mitochondrial ATP-sensitive potassium channels (mitoK_ATP_) and the closure of mitochondrial permeability transition pores (mPTP) (Zuo et al., 2015b). Likewise, in a neonatal hypoxic-ischemic brain injury model, mitoK_ATP_ were involved in HPC-induced neuroprotection, and the administration of a mitoK_ATP_ channel blocker inhibited this protection (Sun et al., 2015). In addition, mPTP inhibitor has been shown to reduce ischemia-reperfusion (I/R)-induced skeletal muscle damage (Garbaisz et al., 2014). However, the protective effects of HPC and the underlying mechanisms have not been investigated in an aged model of COPD.

Chronic obstructive pulmonary diseases is a late-onset disease primarily found in middle aged and elderly population (Peruzza et al., 2003). It was estimated that 16% of individuals at an age over 65 are affected by COPD. Moreover, COPD that occurs at a later age is linked with significantly reduced quality of life and a higher morbidity (Peruzza et al., 2003). However, there is currently no research that explores the potential benefits of HPC in elderly COPD patients. Therefore, in this study, we aimed to investigate HPC effects in an aged COPD-like mouse model during reoxygenation. We used mice with overexpression of pulmonary tumor necrosis factor-α (TNF-α; Tg^+^) that develop emphysematous alterations (Lundblad et al., 2005; Fujita et al., 2016). Multiple studies have indicated that TNF-α overexpression is necessary to promote the disease (Churg et al., 2004; Lundblad et al., 2005). Elevated expression of pulmonary TNF-α also lead to skeletal muscle dysfunction and increased ROS generation in contracting muscles, as commonly seen in COPD (Man et al., 2009; Zuo et al., 2014b). We hypothesize that HPC attenuates muscle damage during reoxygenation by strengthening the Tg^+^ diaphragm muscle through intracellular signaling cascades that involve ROS and certain mitochondrial channels.

## Materials and Methods

### Animals and Muscle Strip Preparation

**FIGURE 1:**
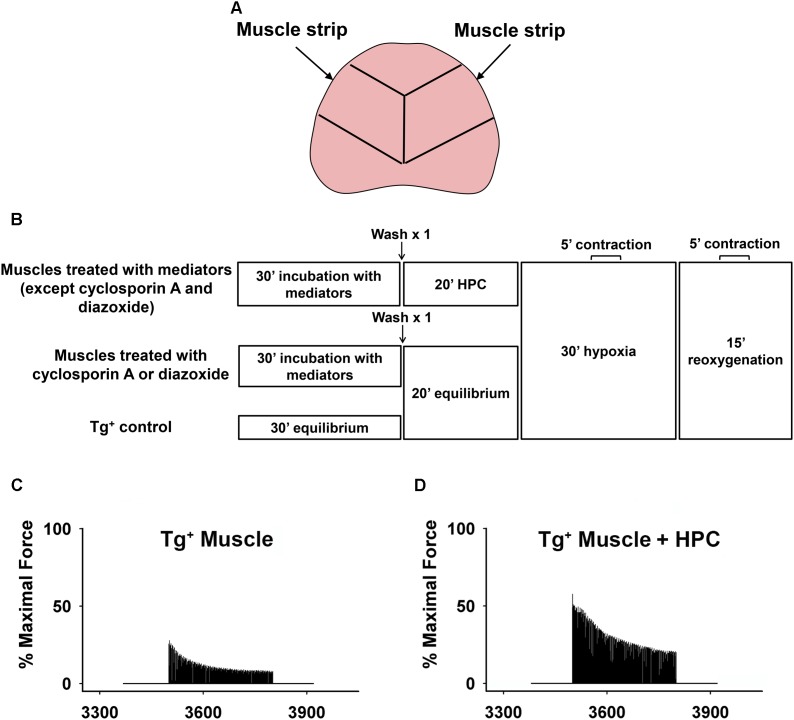
**(A)** Schematic showing that two muscle strips were dissected out from each mouse diaphragm. Lines represent the position where the diaphragm was cut to make muscle strips. **(B)** Schematic showing hypoxia-reoxygenation protocol. Representative curves demonstrating the percentage of maximal force during reoxygenation. **(C)** Tg^+^ muscle vs. **(D)** Tg^+^ muscle + HPC.

Adult transgenic mice overexpressing pulmonary TNF-α with the control of surfactant protein (SP)-C were produced by crossing male SP-C/TNF-α Tg^+^ (a generous gift from Dr. I. Mark Olfert, West Virginia University School of Medicine) mice with female C57BL/6 mice followed by PCR screening to confirm genotype (Thomson et al., 2012; Zuo et al., 2014b). All procedures involving animals were completed in strict accordance with the Guide for the Care and Use of Laboratory Animals of the National Institutes of Health, and the protocol was approved by The Ohio State University Institutional Animal Care and Use Committee. Male Tg^+^ mice aged an average of ∼20 mo (∼20–30 g body weight) were intraperitoneally anesthetized with a mixture of ketamine (80 mg/kg) and xylazine (10 mg/kg). The diaphragm was quickly removed and two muscle strips (∼0.5 cm wide) were dissected out from each leaflet of the diaphragm. Muscle strips were obtained from a similar position of each diaphragm to ensure a similar muscle fiber-type distribution between the muscle strips (Figure 1A). The isolated muscle strips were mounted in a contracting chamber filled with oxygenated Ringer’s solution (121 mM NaCl, 21 mM NaHCO_3_, 11.5 mM glucose, 5.9 mM KCl, 2.0 mM CaCl_2_, 1.2 mM NaH_2_PO_4_, 1.0 mM MgCl_2_, and 0.9 mM Na_2_SO_4_; 21°C; pH 7.4) (Zuo et al., 2015b). Older mice were used in this study in order to simulate elderly or severe COPD patients who have suffered from extended periods of breathing difficulties due to diaphragm dysfunction and require the use of supplemental oxygen, granting clinical potentials of HPC in this population.

### Muscle Function Experiment With Mediators

In order to determine potential signaling mediators involved in the HPC pathway during reoxygenation, various inhibitors/activators were administered to muscle strips. The muscle strips were incubated in oxygenated Ringer’s solution with respective inhibitors for 30 min prior to HPC treatment or equilibrium (Zuo et al., 2015b): antioxidants, *N*-acetyl cysteine (NAC; 1 mM; Sigma) and Tiron (O_2_^•−^ scavenger; 1 mM; Sigma) (Mohanraj et al., 1998); PI3K inhibitor (LY294002; 100 μM; EMD Millipore Corporation) (Dogra et al., 2006); Akt inhibitor (MK-2206; 50 μM; Selleck Chemicals) (Sharma et al., 2012); ERK inhibitor (PD98059; 100 μM; Promega) (Ryder et al., 2000a); mPTP opener, carboxyatractyloside (CAR; 50 μM; Sigma) (Malekova et al., 2007); mPTP opening inhibitor, cyclosporin A (100 μM; Cell Signaling Technology) (Pottecher et al., 2013); K_ATP_ channel opening inhibitor, glibenclamide (100 μM; Sigma) (Flagg et al., 2010); and mitoK_ATP_ channel opener, diazoxide (100 μM; Sigma) (Flagg et al., 2010) at 21°C. All inhibitors/mediators were prepared in DMSO except for antioxidants, NAC and Tiron, which were dissolved in deionized water according to manufacture’s protocols. After 30 min of incubation, muscle strips were washed once and preserved in fresh Ringer’s solution without the addition of drugs. The muscle strips were then subjected to HPC (alternating 2-min exposures of 95 N_2_-5% CO_2_ and 95% O_2_-5% CO_2_ for five times; 20 min total), in accordance with the previous study (Roberts et al., 2013). These acute low oxygen (95% N_2_) and hyperoxic (95% O_2_ ∼722 mmHg) *ex vivo* conditions were utilized to ensure sufficient cellular levels of hypoxia and reoxygenation in our settings, which could have been affected by diffusion distance in the mouse diaphragm due to its thickness and potential leakage of gas from the non-sealed contraction chamber (Zuo et al., 2015b). For the two groups treated either with cyclosporin A or diazoxide, muscles were equilibrated in Ringer’s solution bubbled with 95% O_2_-5% CO_2_ for 20 min instead of HPC. The Tg^+^ control group followed the same protocol in the absence of inhibitor (replaced by Ringer’s solution) or HPC treatment (Figure 1B).

The muscle strips were then exposed to 30-min hypoxia (95 N_2_-5% CO_2_) followed by 15-min reoxygenation (95% O_2_-5% CO_2_). Continuous stimulation of muscle strips for 5 min to measure contractile force were introduced during 15–20 min of the hypoxic and 10–15 min of the reoxygenation periods (Figure 1B). Ringer’s solutions were maintained at 37°C (normal human body temperature) during all muscle contraction measurements. Measured force was normalized by the maximal contraction during the equilibrium before HPC treatment and after 30-min incubation with mediators in order to minimize any potential confounding effects introduced by individual mice variations (Figure 1B). A myograph (model 800MS; Danish Myo Technology, Aarhus, Denmark) was used to measure muscle function, as described in the previous studies (Mohanraj et al., 1998; Zuo et al., 2011). Prior to initial testing, each muscle strip was stretched to attain maximal force of contractions using a mobile lever arm. Muscle contractile force was determined by a stationary force transducer (force range 0–1,600 mN), which was calibrated to convert electrical voltage to tension development per gram. The muscle was electrically stimulated (S48 stimulator, Grass Technologies, West Warwick, RI) with square-wave electrical pulses (0.2-ms pulse period, 250-ms train duration, 70 Hz, at 30 V). A converter (model ML826; AD Instruments, Colorado Springs, CO, United states) then translated these tetanic contractions into digital data for the interpretation in LabChart 7.3.7 analyzing software.

### Statistics

One-way ANOVA was used to analyze the data for statistical significance, and was expressed sequentially as means +/− SE (IBM SPSS Statistics 21). Post-ANOVA contrast analyses from IBM SPSS software was used to identify differences between treatments. *p* < 0.05 was considered statistically significant for paired or independent sample *t*-tests, in comparison of same group samples or differing group samples, respectively. A sample size of five mice per group was determined using G^∗^Power version 3.1.6 that can provide 80% power to detect an effect size of 0.25 at a significance level of 0.05 based upon our previous data on wild-type mice.

## Results

**FIGURE 2:**
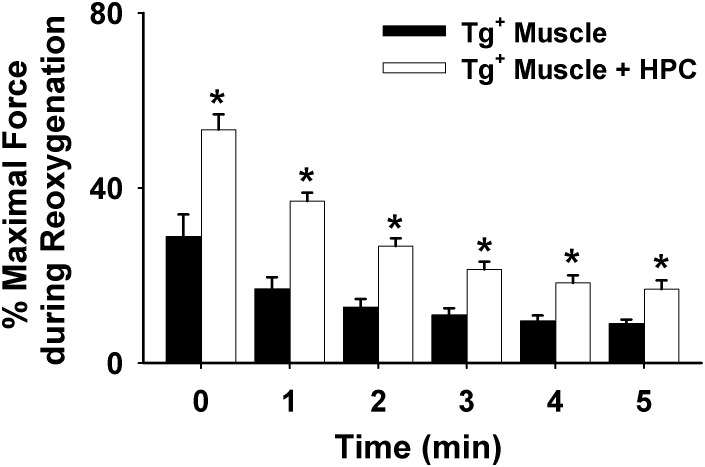
Averaged data illustrating the force decline during the 5-min contractile period under reoxygenation from muscles of Tg^+^ mouse (*n* = 6) vs. Tg^+^ mouse + HPC (*n* = 5). ^∗^Significant different from Tg^+^, *p* < 0.05.

As shown in Figures 1C,D, HPC treatment considerably reduced diaphragm muscle function decline during reoxygenation in Tg^+^ mice. Further, the percentage of maximal force in the Tg^+^ + HPC group was markedly higher than the Tg^+^ control group at each subsequent time point (0–5 min) during reoxygenation, suggesting that HPC improves muscle function (*n* = 5, *p* < 0.01 for 1–5 min contraction; Figure 2).

**FIGURE 3:**
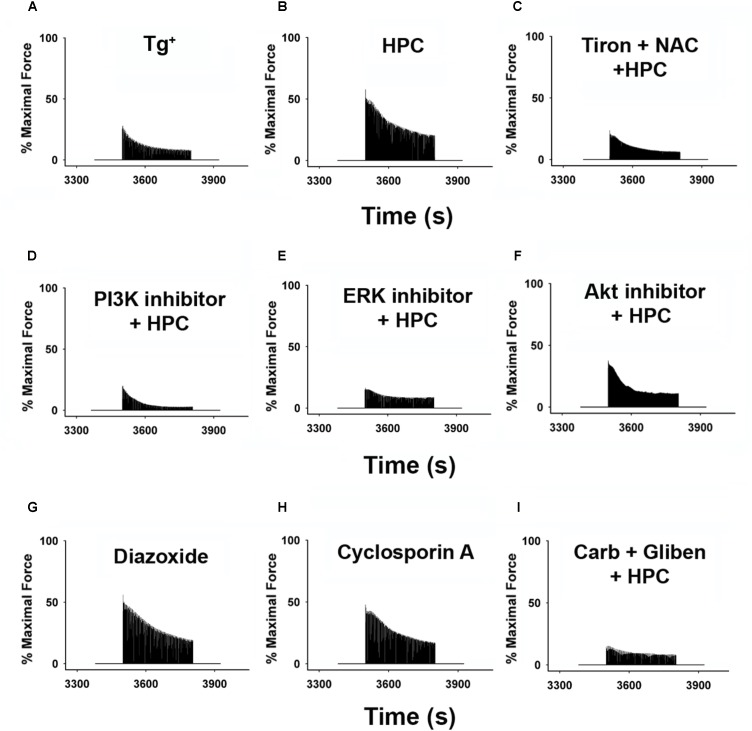
Representative reoxygenation curves showing percentage of force compared with baseline contraction of muscles incubated with respective inhibitors/activators. Data represent approximate average contractile curve for each condition. **(A)** Tg^+^ control; *n* = 6. **(B)** HPC; *n* = 5. **(C)** 1 mM Tiron (antioxidant) + 1 mM NAC (antioxidant) + HPC; *n* = 5. **(D)** 100 μM LY294002 (PI3K inhibitor) + HPC; *n* = 6. **(E)** 50 μM MK-2206 (Akt inhibitor) + HPC; *n* = 5. **(F)** 100 μM PD98059 (ERK inhibitor) + HPC; *n* = 5. **(G)** 100 μM diazoxide (K_ATP_ channel); *n* = 5. **(H)** 100 μM cyclosporin A (mPTP opening inhibitor); *n* = 5. **(I)** 100 μM gliben (K_ATP_ channel opening inhibitor) + 50 μM carb (mPTP opener) + HPC; *n* = 7. AKT, protein kinase B; Carb, carboxyatractyloside; ERK, extracellular signal-regulated kinases; Gliben, glibenclamide; HPC, hypoxic preconditioning; K_ATP_, mitochondrial ATP-sensitive potassium channel; mPTP, mitochondrial permeability transition pore; NAC, *N*-acetyl cysteine; PI3K, phosphoinositide 3-kinase.

Representative reoxygenation curves displaying the percentage of maximal contractile force with respective inhibitors/activators are depicted in Figure 3. The effects of HPC in improving muscle function were obliterated after incubation with (1) antioxidants (Tiron and NAC) in Figure 3C; (2) non-mitochondrial inhibitors (PI3K inhibitor, LY294002; ERK inhibitor, PD98059; AKT inhibitor, MK-2206) in Figures 3D–F; and (3) mitochondrial mediators (glibenclamide + carboxyatractyloside) in Figure 3I. Muscle strips incubated with either diazoxide or cyclosporin A individually demonstrated improved contraction force comparable to the HPC curve (Figures 3G,H, respectively). Diazoxide and cyclosporin A are potent mitoK_ATP_ channel opener and mPTP closer, respectively, which are both commonly used in studies investigating the signaling cascade underlying protection associated with ischemic preconditioning (Pain et al., 2000; Hausenloy et al., 2004; Hanley and Daut, 2005). Similarly, our previous study using diazoxide or cyclosporin A reported that HPC protective effects involve the closure of mPTP and the opening of mitoK_ATP_ channels in wild-type muscles (Zuo et al., 2015b). Our data show that both diazoxide and cyclosporin A treatment generates similar beneficial effects to that from HPC, suggesting the potential involvement of mitoK_ATP_ channel and mPTP in HPC-initiated effects.

**FIGURE 4:**
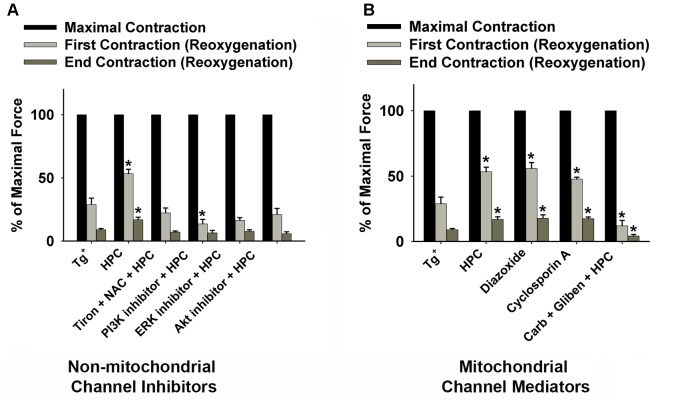
Grouped data displaying the decline in diaphragmatic force (fatigue) during the 15-min reoxygenation period from Tg^+^ control (*n* = 6); HPC (*n* = 5); **(A)** non-mitochondrial channel inhibitors [1 mM Tiron (antioxidant) + 1 mM NAC (antioxidant) + HPC (*n* = 5); 100 μM LY294002 (PI3K inhibitor) + HPC (*n* = 6); 50 μM MK-2206 (Akt inhibitor) + HPC (*n* = 5); 100 μM PD98059 (ERK inhibitor) + HPC (*n* = 5)]; and **(B)** mitochondrial channel mediators [100 μM diazoxide (K_ATP_ channel, *n* = 5); 100 μM cyclosporin A (mPTP opening inhibitor, *n* = 5); 100 μM gliben (K_ATP_ channel opening inhibitor) + 50 μM carb (mPTP opener) + HPC (*n* = 7)]. ^∗^Significantly different from control (*p* < 0.05). Baseline contraction forces were measured before HPC treatment. AKT, protein kinase B; Carb, carboxyatractyloside; ERK, extracellular signal-regulated kinases; Gliben, glibenclamide; HPC, hypoxic preconditioning; K_ATP_, mitochondrial ATP-sensitive potassium channel; mPTP, mitochondrial permeability transition pore; NAC, *N*-acetyl cysteine; PI3K, phosphoinositide 3-kinase.

Grouped data summarizing the percentage of the maximal force at initial and end contraction during reoxygenation with different inhibitors or mediators are displayed in Figures 4A,B. The results confirmed that the Tg^+^ muscles can be significantly strengthened during reoxygenation after treating with HPC (*n* = 5; *p* = 0.004 for the 1st contraction; *p* = 0.005 for the end contraction), diazoxide *n* = 5; *p* = 0.004 for the 1st contraction; *p* = 0.006 for the end contraction), or cyclosporin A (*n* = 5; *p* = 0.01 for the 1st contraction; *p* = 0.000 for the end contraction). In Figure 4B, exposure to either diazoxide or cyclosporin A alone produced similar significance in muscle function as HPC. The normalized initial and end contractile forces increased compared to the control (n = 5; *p* = 0.004 for the 1st contraction; *p* = 0.005 for the end contraction). Non-mitochondrial signaling inhibitors and mitochondrial mediators (glibenclamide + carboxyatractyloside) diminished HPC-induced improvement in muscle function (Figure 4). Notably, muscle treated with glibenclamide + carboxyatractyloside resulted in contractile force significantly lower than control (Figure 4B; *n* = 7, *p* = 0.024 for the 1st contraction; *p* = 0.009 for the end contraction).

## Discussion

Our *ex vivo* results show that diaphragm muscle function during reoxygenation was significantly improved after HPC treatment in aged Tg^+^ mice. Using specific inhibitors and mitochondrial mediators, we have identified the potential HPC pathways, which may involve the activation of ROS, PI3K, Akt, ERK, as well as the triggering of mitoK_ATP_ channel opening and mPTP closure.

### Role of ROS in HPC

Our previous studies on wild-type mice have similarly shown that the diaphragmatic function can be significantly improved by HPC during reoxygenation (Zuo and Re, 2014; Zuo et al., 2015c). The development of muscle fatigue is known to be associated with ATP depletion, metabolic by-product (e.g., H^+^ and phosphate) accumulation, decreased myofibrillar Ca^2+^ sensitivity, and oxidative stress (Powers et al., 2011; Grassi et al., 2015; Debold, 2016). Interestingly, ROS play biphasic roles in skeletal muscle function and are considered to be the major mediators of preconditioning (Zuo et al., 2013a; He et al., 2016). Specifically, studies have shown that the elevation of xanthine oxidase (XO) contributes greatly to reoxygenation-associated ROS accumulation (Ozyurt et al., 2006; Moylan and Reid, 2007). An overproduction of ROS is considered as one of the contributors of reduced muscle function and it results in lipid peroxidation and oxidative alteration of cellular proteins thereby leading to reoxygenation injury (Reid et al., 1992; Li and Jackson, 2002; Zuo et al., 2013b). Optimal levels of ROS are involved in the intracellular adaptations and protection of various tissues by preconditioning (Costa et al., 2013; Zuo et al., 2013a). Our previous data on wild-type mice showed that HPC abolished the increase in ROS production during hypoxia and reoxygenation (Zuo et al., 2014a, 2015b). It is likely that this reduced level of ROS plays a key role in triggering the protective effect of HPC as well as lowering oxidative damage during reoxygenation. In the present study, ROS scavenging by the exogenous antioxidants, Tiron and NAC, attenuated the improved muscle function offered by HPC (Figures 3C, 4A), further implying the involvement of ROS in HPC protection.

### PI3K-AKT-ERK Pathways in HPC

Moreover, in Tg^+^ mice models, diaphragmatic muscles treated with any of the cell signaling inhibitors of PI3K, Akt, and ERK showed diminished HPC effects (Figure 4A). Consistent findings were reported in other models such as cardiac progenitor cells, which suggest the pivotal role of PI3K, Akt, or ERK in hypoxic protection. For example, Xu et al. (2016) observed that LY294002 (PI3K inhibitor) eliminates the pro-survival effect of HPC *in vitro*. Furthermore, in our study, 30-min incubation with the mediators did not alter the maximal muscle contractile force (data not shown), which suggests that the mediators used in our experiments did not have marked influence on muscle function in non-HPC treated muscles. In addition, Akt and ERK activation is beneficial to skeletal muscle by inducing increased ATP production, mitochondrial biogenesis, and functional adaptation, which is consistent with our result of HPC (Hawley, 2002; Berdeaux and Stewart, 2012). Muscle contraction may increase glucose uptake via the activation of PI3K/Akt cascades in an insulin-dependent manner (Zhou and Dohm, 1997; Soos et al., 2001; Rose and Richter, 2005). In the absence of insulin, PI3K/Akt signaling was probably kept deactivated. However, HPC may activate PI3K/AKT independent of insulin, which stimulate glucose uptake (Ryder et al., 2000b) or regulate downstream mitochondrial channels to enhance the muscle function. Previous studies on the ERK involvement in muscle contraction have been focused on muscle adaptive responses to exercise or hypoxia via the upregulation of nuclear factor-κB (NF-κB) (Osorio-Fuentealba et al., 2009; Assaly et al., 2011; He et al., 2016). Our data show that ERK inhibition reduced HPC-treated muscle function during reoxygenation, which suggests an acute effect mediated by ERK activation in HPC-induced muscle cell protection.

### MitoK_ATP_ and MPTP in HPC

Our previous study showed that HPC induces both the closure of mPTP and the opening of mitoK_ATP_ channels in wild-type muscles (Zuo et al., 2015b). Similar results were observed in Tg^+^ muscles. Research has shown that abrupt mPTP opening, in response to oxidative stress and mitochondrial Ca^2+^ overload, is evident at the onset of reperfusion (Husainy et al., 2012; Garbaisz et al., 2014). The application of carboxyatractyloside to prevent mPTP closure may exacerbate the mPTP-induced cell injury. This may explain why HPC muscles incubated with carboxyatractyloside and glibenclamide showed significant lower muscle function than control muscles during reoxygenation (Figure 4B). The suppression of mPTP is crucial to retain mitochondrial integrity and prevent cell death (Husainy et al., 2012; Chen et al., 2015). Moreover, previous evidence has shown that mitoK_ATP_ channel opening can be significantly activated in ischemic heart, indicating their critical roles in muscle protection under stress (Zhuo et al., 2005). Our data suggests that HPC may be able to enhance such protective effects associated with mitoK_ATP_ channel openings. ROS generated due to the activation of mitoK_ATP_ channels are reported to trigger preconditioning (Lebuffe et al., 2003). It was reported that the reduction of mitoK_ATP_ channel opening is associated with the extent of fatigue (Garcia et al., 2009). In addition, mitoK_ATP_ channel openings have been shown to lead to K^+^-specific mitochondrial depolarization and swelling of de-energized mitochondria in rat skeletal muscles, thereby modulating oxidative phosphorylation (Pottecher et al., 2013).

### Limitations

There are a few limitations in this study. First, due to the need for continuous contraction, the diaphragm has high oxidative enzyme activities, giving it greater resistance to fatigue compared to other respiratory muscles (Polla et al., 2004). Despite altered fiber distribution and slow energy consumption, the diaphragm of COPD subjects produces similar isometric forces as non-COPD subjects, suggesting that the active cross-bridges generate greater force than those of control muscle (Polkey et al., 1996; Stubbings et al., 2008). Second, overexpressed TNF-α can elevate ROS production, likely contributing to HPC-related molecular alterations (Machida et al., 2003). Thus, our future studies will directly determine the correlations between TNF-α levels and HPC effects. Third, diaphragm was kept at Ringer’s solution bubbled with 95% O_2_ after isolation. However, this operation can cause additional reoxygenation-related muscle damage. The diaphragmatic muscles were stimulated for 5 min during both hypoxic and reoxygenation periods. This tetanic contraction during hypoxia is energetically expensive for muscles and can lead to additional fatigue or injury, thus affecting muscle performance during the following reoxygenation. All these limitations can possibly create some bias in the evaluation of reoxygenation-associated muscle function.

## Perspectives and Significance

Little research has been focused on HPC or non-pharmacological treatments of respiratory skeletal muscle during reperfusion injuries and in the context of COPD in elderly. Here for the first time, we demonstrate that aged Tg^+^ (COPD-like) skeletal muscle which has undergone HPC sustains muscle force production and exhibits improved muscle function during reoxygenation. A possible signaling pathway involved in HPC protection has been proposed. Identifying the interaction between ROS, PI3K, Akt, ERK, and mitochondrial channels in HPC will be useful in developing pharmacological treatment that can enhance HPC effects. However, additional studies are needed to fully evaluate the clinical feasibility and effectiveness of HPC in different models.

## Author Contributions

LZ conceived and designed the research. C-CC and TZ performed the experiments. C-CC, TZ, and LZ analyzed the data and drafted the manuscript. C-CC and LZ interpreted the results of experiments. TZ prepared the figures. C-CC, TZ, IO, and LZ edited and revised the manuscript. IO provided the male SP-C/TNF-α Tg^+^ mice. C-CC, TZ, IO, and LZ approved the final version of the manuscript.

## Conflict of Interest Statement

The authors declare that the research was conducted in the absence of any commercial or financial relationships that could be construed as a potential conflict of interest.

## References

[B1] Assaly R. De Tassigny A. D. Jacquin S. Mompied C. Berdeaux A. Morin D. (2011). Oxidative stress during hypoxia is necessary to induce mitochondrial permeability transition pore opening and cell death during hypoxia/reoxygenation in adult cardiomyocytes. *Fund Clin. Pharmacol.* 25 46–47. 10.1016/j.ejphar.2011.11.036 22173126

[B2] Berdeaux R. Stewart R. (2012). cAMP signaling in skeletal muscle adaptation: hypertrophy, metabolism, and regeneration. *Am. J. Physiol. Endocrinol. Metab.* 303 E1–E17. 10.1152/ajpendo.00555.2011 22354781 PMC3404564

[B3] Cazzola M. Bettoncelli G. Sessa E. Cricelli C. Biscione G. (2010). Prevalence of comorbidities in patients with chronic obstructive pulmonary disease. *Respiration* 80 112–119. 10.1159/000281880 20134148

[B4] Chen Y. Liu J. Zheng Y. Wang J. Wang Z. Gu S. (2015). Uncoupling protein 3 mediates H_2_O_2_ preconditioning-afforded cardioprotection through the inhibition of MPTP opening. *Cardiovasc. Res.* 105 192–202. 10.1093/cvr/cvu256 25514931

[B5] Churg A. Wang R. D. Tai H. Wang X. S. Xie C. S. Wright J. L. (2004). Tumor necrosis factor-alpha drives 70% of cigarette smoke-induced emphysema in the mouse. *Am. J. Respir. Crit. Care Med.* 170 492–498. 10.1164/rccm.200404-5110C 15184206

[B6] Costa J. F. Fontes-Carvalho R. Leite-Moreira A. F. (2013). Myocardial remote ischemic preconditioning: from pathophysiology to clinical application. *Rev. Port. Cardiol.* 32 893–904. 10.1016/j.repc.2013.02.012 24120469

[B7] Debold E. P. (2016). Decreased myofilament calcium sensitivity plays a significant role in muscle fatigue. *Exerc. Sport Sci. Rev.* 44 144–149. 10.1249/JES.0000000000000089 27526194

[B8] Debska G. Kicinska A. Skalska J. Szewczyk A. May R. Elger C. E. (2002). Opening of potassium channels modulates mitochondrial function in rat skeletal muscle. *Biochim. Biophys. Acta* 1556 97–105. 12460666 10.1016/s0005-2728(02)00340-7

[B9] Dogra C. Changotra H. Wergedal J. E. Kumar A. (2006). Regulation of phosphatidylinositol 3-kinase (PI3K)/Akt and nuclear factor-kappa B signaling pathways in dystrophin-deficient skeletal muscle in response to mechanical stretch. *J. Cell. Physiol.* 208 575–585. 10.1002/jcp.20696 16741926

[B10] Flagg T. P. Enkvetchakul D. Koster J. C. Nichols C. G. (2010). Muscle KATP channels: recent insights to energy sensing and myoprotection. *Physiol. Rev.* 90 799–829. 10.1152/physrev.00027.2009 20664073 PMC3125986

[B11] Fujita M. Ouchi H. Ikegame S. Harada E. Matsumoto T. Uchino J. (2016). Critical role of tumor necrosis factor receptor 1 in the pathogenesis of pulmonary emphysema in mice. *Int. J. Chron. Obstruct. Pulmon. Dis.* 11 1705–1712. 10.2147/COPD.S108919 27555760 PMC4968668

[B12] Garbaisz D. Turoczi Z. Aranyi P. Fulop A. Rosero O. Hermesz E. (2014). Attenuation of skeletal muscle and renal injury to the lower limb following ischemia-reperfusion using mPTP inhibitor NIM-811. *PLoS One* 9:e101067. 10.1371/journal.pone.0101067 24968303 PMC4072765

[B13] Garcia M. C. Hernandez A. Sanchez J. A. (2009). Role of mitochondrial ATP-sensitive potassium channels on fatigue in mouse muscle fibers. *Biochem. Biophys. Res. Commun.* 385 28–32. 10.1016/j.bbrc.2009.05.019 19427835

[B14] Grassi B. Rossiter H. B. Zoladz J. A. (2015). Skeletal muscle fatigue and decreased efficiency: two sides of the same coin? *Exerc. Sport Sci. Rev.* 43 75–83. 10.1249/JES.0000000000000043 25688762

[B15] Hanley P. J. Daut J. (2005). K(ATP) channels and preconditioning: a re-examination of the role of mitochondrial K(ATP) channels and an overview of alternative mechanisms. *J. Mol. Cell. Cardiol.* 39 17–50. 10.1016/j.yjmcc.2005.04.002 15907927

[B16] Hausenloy D. Wynne A. Duchen M. Yellon D. (2004). Transient mitochondrial permeability transition pore opening mediates preconditioning-induced protection. *Circulation* 109 1714–1717. 10.1161/01.CIR.0000126294.81407.7D 15066952

[B17] Hawley J. A. (2002). Adaptations of skeletal muscle to prolonged, intense endurance training. *Clin. Exp. Pharmacol. Physiol.* 29 218–222. 11906487 10.1046/j.1440-1681.2002.03623.x

[B18] He F. Li J. Liu Z. Chuang C. C. Yang W. Zuo L. (2016). Redox mechanism of reactive oxygen species in exercise. *Front. Physiol.* 7:486 10.3389/fphys.2016.00486 PMC5097959 27872595

[B19] Huang C. L. Nguyen P. A. Kuo P. L. Iqbal U. Hsu Y. H. E. Jian W. S. (2013). Influenza vaccination and reduction in risk of ischemic heart disease among chronic obstructive pulmonary elderly. *Comput. Methods Programs Biomed.* 111 507–511. 10.1016/j.cmpb.2013.05.006 23769164

[B20] Husainy M. A. Dickenson J. M. Galinanes M. (2012). The MPTP status during early reoxygenation is critical for cardioprotection. *J. Surg. Res.* 174 62–72. 10.1016/j.jss.2010.11.879 21316705

[B21] Kim H. C. Mofarrahi M. Hussain S. N. (2008). Skeletal muscle dysfunction in patients with chronic obstructive pulmonary disease. *Int. J. Chron. Obstruct. Pulmon. Dis.* 3 637–658. 19281080 10.2147/copd.s4480 PMC2650609

[B22] Lebuffe G. Schumacker P. T. Shao Z. H. Anderson T. Iwase H. Vanden Hoek T. L. (2003). ROS and NO trigger early preconditioning: relationship to mitochondrial KATP channel. *Am. J. Physiol. Heart. Circ. Physiol.* 284 H299–H308. 10.1152/ajpheart.00706.2002 12388274

[B23] Li C. Jackson R. M. (2002). Reactive species mechanisms of cellular hypoxia-reoxygenation injury. *Am. J. Physiol. Cell Physiol.* 282 C227–C241. 10.1152/ajpcell.00112.2001 11788333

[B24] Lundblad L. K. Thompson-Figueroa J. Leclair T. Sullivan M. J. Poynter M. E. Irvin C. G. (2005). Tumor necrosis factor-alpha overexpression in lung disease: a single cause behind a complex phenotype. *Am. J. Respir. Crit. Care Med.* 171 1363–1370. 10.1164/rccm.200410-1349OC 15805183 PMC2718479

[B25] Machida Y. Kubota T. Kawamura N. Funakoshi H. Ide T. Utsumi H. (2003). Overexpression of tumor necrosis factor-alpha increases production of hydroxyl radical in murine myocardium. *Am. J. Physiol. Heart Circ. Physiol.* 284 H449–H455. 10.1152/ajpheart.00581.2002 12388222

[B26] Malekova L. Kominkova V. Ferko M. Stefanik P. Krizanova O. Ziegelhoffer A. (2007). Bongkrekic acid and atractyloside inhibits chloride channels from mitochondrial membranes of rat heart. *Biochim. Biophys. Acta* 1767 31–44. 10.1016/j.bbabio.2006.10.004 17123460

[B27] Man W. D. Kemp P. Moxham J. Polkey M. I. (2009). Skeletal muscle dysfunction in COPD: clinical and laboratory observations. *Clin. Sci.* 117 251–264. 10.1042/CS20080659 19681758

[B28] Mohanraj P. Merola A. J. Wright V. P. Clanton T. L. (1998). Antioxidants protect rat diaphragmatic muscle function under hypoxic conditions. *J. Appl. Physiol.* 84 1960–1966. 9609790 10.1152/jappl.1998.84.6.1960

[B29] Moylan J. S. Reid M. B. (2007). Oxidative stress, chronic disease, and muscle wasting. *Muscle Nerve* 35 411–429. 10.1002/mus.20743 17266144

[B30] Orozco-Levi M. Lloreta J. Minguella J. Serrano S. Broquetas J. M. Gea J. (2001). Injury of the human diaphragm associated with exertion and chronic obstructive pulmonary disease. *Am. J. Respir. Crit. Care Med.* 164 1734–1739. 10.1164/ajrccm.164.9.2011150 11719318

[B31] Osorio-Fuentealba C. Valdes J. A. Riquelme D. Hidalgo J. Hidalgo C. Carrasco M. A. (2009). Hypoxia stimulates via separate pathways ERK phosphorylation and NF-kappaB activation in skeletal muscle cells in primary culture. *J. Appl. Physiol.* 106 1301–1310. 10.1152/japplphysiol.91224.2008 19179647

[B32] Ozyurt B. Iraz M. Koca K. Ozyurt H. Sahin S. (2006). Protective effects of caffeic acid phenethyl ester on skeletal muscle ischemia-reperfusion injury in rats. *Mol. Cell. Biochem.* 292 197–203. 10.1007/s11010-006-9232-5 16786192

[B33] Pain T. Yang X. M. Critz S. D. Yue Y. Nakano A. Liu G. S. (2000). Opening of mitochondrial K(ATP) channels triggers the preconditioned state by generating free radicals. *Circ. Res.* 87 460–466. 10988237 10.1161/01.res.87.6.460

[B34] Peruzza S. Sergi G. Vianello A. Pisent C. Tiozzo F. Manzan A. (2003). Chronic obstructive pulmonary disease (COPD) in elderly subjects: impact on functional status and quality of life. *Respir. Med.* 97 612–617. 10.1053/rmed.2003.1488 12814144

[B35] Polkey M. I. Kyroussis D. Hamnegard C. H. Mills G. H. Green M. Moxham J. (1996). Diaphragm strength in chronic obstructive pulmonary disease. *Am. J. Respir. Crit. Care Med.* 154 1310–1317. 10.1164/ajrccm.154.5.8912741 8912741

[B36] Polla B. D’antona G. Bottinelli R. Reggiani C. (2004). Respiratory muscle fibres: specialisation and plasticity. *Thorax* 59 808–817. 10.1136/thx.2003.009894 15333861 PMC1747126

[B37] Pottecher J. Guillot M. Belaidi E. Charles A. L. Lejay A. Gharib A. (2013). Cyclosporine A normalizes mitochondrial coupling, reactive oxygen species production, and inflammation and partially restores skeletal muscle maximal oxidative capacity in experimental aortic cross-clamping. *J. Vasc. Surg.* 57 1100–1108.e2. 10.1016/j.jvs.2012.09.020 23332985

[B38] Powers S. K. Ji L. L. Kavazis A. N. Jackson M. J. (2011). Reactive oxygen species: impact on skeletal muscle. *Compr. Physiol.* 1 941–969. 10.1002/cphy.c100054 23737208 PMC3893116

[B39] Reid M. B. Haack K. E. Franchek K. M. Valberg P. A. Kobzik L. West M. S. (1992). Reactive oxygen in skeletal muscle. I. Intracellular oxidant kinetics and fatigue in vitro. *J. Appl. Physiol.* 73 1797–1804. 1474054 10.1152/jappl.1992.73.5.1797

[B40] Roberts W. J. Yousif M. K. Hallman A. H. Zuo L. (2013). Hypoxic preconditioning reduces reoxygenation injuries via PI3K in respiratory muscle. *Med. Sci. Sport Exerc.* 45 295–295.

[B41] Rose A. J. Richter E. A. (2005). Skeletal muscle glucose uptake during exercise: how is it regulated? *Physiology* 20 260–270. 10.1152/physiol.00012.2005 16024514

[B42] Ryder J. W. Fahlman R. Wallberg-Henriksson H. Alessi D. R. Krook A. Zierath J. R. (2000a). Effect of contraction on mitogen-activated protein kinase signal transduction in skeletal muscle. Involvement Of the mitogen- and stress-activated protein kinase 1. *J. Biol. Chem.* 275 1457–1462. 10.1074/jbc.275.2.1457 10625698

[B43] Ryder J. W. Yang J. Galuska D. Rincon J. Bjornholm M. Krook A. (2000b). Use of a novel impermeable biotinylated photolabeling reagent to assess insulin- and hypoxia-stimulated cell surface GLUT4 content in skeletal muscle from type 2 diabetic patients. *Diabetes Metab. Res. Rev.* 49 647–654. 10.2337/diabetes.49.4.647 10871204

[B44] Sharma N. Arias E. B. Sequea D. A. Cartee G. D. (2012). Preventing the calorie restriction-induced increase in insulin-stimulated Akt2 phosphorylation eliminates calorie restriction’s effect on glucose uptake in skeletal muscle. *Biochim. Biophys. Acta* 1822 1735–1740. 10.1016/j.bbadis.2012.07.012 22846604 PMC3444632

[B45] Soos M. A. Jensen J. Brown R. A. O’rahilly S. Shepherd P. R. Whitehead J. P. (2001). Class II phosphoinositide 3-kinase is activated by insulin but not by contraction in skeletal muscle. *Arch. Biochem. Biophys.* 396 244–248. 10.1006/abbi.2001.2587 11747303

[B46] Steinbacher P. Eckl P. (2015). Impact of oxidative stress on exercising skeletal muscle. *Biomolecules* 5 356–377. 10.3390/biom5020356 25866921 PMC4496677

[B47] Stoller J. K. Panos R. J. Krachman S. Doherty D. E. Make B. Long-Term Oxygen Treatment Trial Research Group. (2010). Oxygen therapy for patients with COPD: current evidence and the long-term oxygen treatment trial. *Chest* 138 179–187. 10.1378/chest.09-2555 20605816 PMC2897694

[B48] Stubbings A. K. Moore A. J. Dusmet M. Goldstraw P. West T. G. Polkey M. I. (2008). Physiological properties of human diaphragm muscle fibres and the effect of chronic obstructive pulmonary disease. *J. Physiol.* 586 2637–2650. 10.1113/jphysiol.2007.149799 18372305 PMC2464347

[B49] Sun H. S. Xu B. Chen W. Xiao A. Turlova E. Alibraham A. (2015). Neuronal K(ATP) channels mediate hypoxic preconditioning and reduce subsequent neonatal hypoxic-ischemic brain injury. *Exp. Neurol.* 263 161–171. 10.1016/j.expneurol.2014.10.003 25448006

[B50] Tang K. Murano G. Wagner H. Nogueira L. Wagner P. D. Tang A. (2013). Impaired exercise capacity and skeletal muscle function in a mouse model of pulmonary inflammation. *J. Appl. Physiol.* 114 1340–1350. 10.1152/japplphysiol.00607.2012 23449936 PMC3656431

[B51] Thomson E. M. Williams A. Yauk C. L. Vincent R. (2012). Overexpression of tumor necrosis factor-alpha in the lungs alters immune response, matrix remodeling, and repair and maintenance pathways. *Am. J. Pathol.* 180 1413–1430. 10.1016/j.ajpath.2011.12.020 22322299

[B52] Xu R. Sun Y. Chen Z. Yao Y. Ma G. (2016). Hypoxic preconditioning inhibits hypoxia-induced apoptosis of cardiac progenitor cells via the PI3K/Akt-DNMT1-p53 pathway. *Sci. Rep.* 6:30922. 10.1038/srep30922 27488808 PMC4973228

[B53] Zhou Q. Dohm G. L. (1997). Treadmill running increases phosphatidylinositol 3-kinase activity in rat skeletal muscle. *Biochem. Biophys. Res. Commun.* 236 647–650. 10.1006/bbrc.1997.7028 9245706

[B54] Zhuo M. L. Huang Y. Liu D. P. Liang C. C. (2005). KATP channel: relation with cell metabolism and role in the cardiovascular system. *Int. J. Biochem. Cell Biol.* 37 751–764. 10.1016/j.biocel.2004.10.008 15694835

[B55] Zuo L. Best T. M. Roberts W. J. Diaz P. T. Wagner P. D. (2015a). Characterization of reactive oxygen species in diaphragm. *Acta Physiol.* 213 700–710. 10.1111/apha.12410 25330121

[B56] Zuo L. Pannell B. K. Re A. T. Best T. M. Wagner P. D. (2015b). Po2 cycling protects diaphragm function during reoxygenation via ROS, Akt, ERK, and mitochondrial channels. *Am. J. Physiol. Cell Physiol.* 309 C759–C766. 10.1152/ajpcell.00174.2015 26423578

[B57] Zuo L. Re A. T. Roberts W. J. Zhou T. Hemmelgarn B. T. Pannell B. K. (2015c). Hypoxic preconditioning mitigates diaphragmatic skeletal muscle fatigue during reoxygenation via ROS and ERK signaling. *Med. Sci. Sports Exerc.* 47:330 10.1249/01.mss.0000477319.94793.39

[B58] Zuo L. Diaz P. T. Chien M. T. Roberts W. J. Kishek J. Best T. M. (2014a). PO2 cycling reduces diaphragm fatigue by attenuating ROS formation. *PLoS One* 9:e109884. 10.1371/journal.pone.0109884 25299212 PMC4192541

[B59] Zuo L. Hallman A. H. Roberts W. J. Wagner P. D. Hogan M. C. (2014b). Superoxide release from contracting skeletal muscle in pulmonary TNF-alpha overexpression mice. *Am. J. Physiol. Regul. Integr. Comp. Physiol.* 306 R75–R81. 10.1152/ajpregu.00425.2013 24196666 PMC3921307

[B60] Zuo L. Nogueira L. Hogan M. C. (2011). Effect of pulmonary TNF-alpha overexpression on mouse isolated skeletal muscle function. *Am. J. Physiol. Regul. Integr. Comp. Physiol.* 301 R1025–R1031. 10.1152/ajpregu.00126.2011 21697519 PMC3197448

[B61] Zuo L. Re A. T. (2014). Hypoxic preconditioning protects diaphragm function during reoxygenation via intracellular signaling cascade. *Free Radic. Biol. Med.* 76 S154–S154. 10.1016/j.freeradbiomed.2014.10.574

[B62] Zuo L. Roberts W. J. Tolomello R. C. Goins A. T. (2013a). Ischemic and hypoxic preconditioning protect cardiac muscles via intracellular ROS signaling. *Front. Biol.* 8 305–311. 10.1007/s11515-012-1225-z 25731682

[B63] Zuo L. Shiah A. Roberts W. J. Chien M. T. Wagner P. D. Hogan M. C. (2013b). Low PO2 conditions induce reactive oxygen species formation during contractions in single skeletal muscle fibers. *Am. J. Physiol. Reggul. Integr. Comp. Physiol.* 304 R1009–R1016. 10.1152/ajpregu.00563.2012 23576612 PMC3680753

